# Cerebral small vessel disease among rural-dwelling Chinese older adults: prevalence, distribution, and associated factors

**DOI:** 10.1093/braincomms/fcaf136

**Published:** 2025-04-04

**Authors:** Huisi Zhang, Chunyan Li, Jiafeng Wang, Mingqing Zhao, Ziwei Chen, Qianqian Xie, Tao Gong, Tingting Hou, Yongxiang Wang, Lin Cong, Lenore J Launer, Lin Song, Yifeng Du, Chengxuan Qiu

**Affiliations:** Department of Neurology, Shandong Provincial Hospital Affiliated to Shandong First Medical University, Jinan 250021, Shandong, P.R. China; Department of Neurology, Shandong Provincial Hospital Affiliated to Shandong First Medical University, Jinan 250021, Shandong, P.R. China; Department of Neurology, Shandong Provincial Hospital Affiliated to Shandong First Medical University, Jinan 250021, Shandong, P.R. China; Department of Neurology, Shandong Provincial Hospital Affiliated to Shandong First Medical University, Jinan 250021, Shandong, P.R. China; Department of Neurology, Shandong Provincial Hospital Affiliated to Shandong First Medical University, Jinan 250021, Shandong, P.R. China; Department of Neurology, Shandong Provincial Hospital Affiliated to Shandong First Medical University, Jinan 250021, Shandong, P.R. China; Department of Neurology, Shandong Provincial Hospital Affiliated to Shandong First Medical University, Jinan 250021, Shandong, P.R. China; Department of Neurology, Shandong Provincial Hospital Affiliated to Shandong First Medical University, Jinan 250021, Shandong, P.R. China; Shandong Provincial Clinical Research Center for Neurological Diseases, Jinan 250021, Shandong, P.R. China; Department of Neurology, Shandong Provincial Hospital Affiliated to Shandong First Medical University, Jinan 250021, Shandong, P.R. China; Shandong Provincial Clinical Research Center for Neurological Diseases, Jinan 250021, Shandong, P.R. China; Medical Science and Technology Innovation Center, Shandong First Medical University & Shandong Academy of Medical Sciences, Jinan 250117, Shandong, P.R. China; Department of Neurology, Shandong Provincial Hospital Affiliated to Shandong First Medical University, Jinan 250021, Shandong, P.R. China; Shandong Provincial Clinical Research Center for Neurological Diseases, Jinan 250021, Shandong, P.R. China; Laboratory of Epidemiology and Population Sciences, National Institute on Aging, Bethesda, PO Box 8057, Gaithersburg, MD 20898, USA; Department of Neurology, Shandong Provincial Hospital Affiliated to Shandong First Medical University, Jinan 250021, Shandong, P.R. China; Shandong Provincial Clinical Research Center for Neurological Diseases, Jinan 250021, Shandong, P.R. China; Department of Neurology, Shandong Provincial Hospital Affiliated to Shandong First Medical University, Jinan 250021, Shandong, P.R. China; Shandong Provincial Clinical Research Center for Neurological Diseases, Jinan 250021, Shandong, P.R. China; Medical Science and Technology Innovation Center, Shandong First Medical University & Shandong Academy of Medical Sciences, Jinan 250117, Shandong, P.R. China; Department of Neurology, Shandong Provincial Hospital Affiliated to Shandong First Medical University, Jinan 250021, Shandong, P.R. China; Medical Science and Technology Innovation Center, Shandong First Medical University & Shandong Academy of Medical Sciences, Jinan 250117, Shandong, P.R. China; Department of Neurobiology, Care Sciences and Society, Aging Research Center, Karolinska Institutet-Stockholm University, 171 65 Solna, Sweden

**Keywords:** cerebral small vessel diseases, prevalence, risk factors, epidemiology, population-based study

## Abstract

Epidemiological characteristics of cerebral small vessel disease (cSVD) in the general population, especially among rural older adults, are poorly defined. Here, we reported the prevalence, distribution, and associated factors of cSVD in a rural-dwelling older population in China. This population-based cross-sectional study included 1272 older adults (age ≥60 years; mean age 69.43 years; 58.57% women) who underwent structural brain MRI scans (3.0T) in 2018–2020. MRI markers of cSVD were assessed following the Standards for Reporting Vascular Changes on Neuroimaging-1 criteria. We performed descriptive and regression analyses. The overall prevalence was 20.31% for cerebral microbleeds (CMBs), 26.87% for lacunes, 60.06% for basal ganglia perivascular spaces (PVS), 76.31% for centrum semiovale PVS, 95.74% for deep white matter hyperintensities (WMHs), and 94.17% for periventricular WMHs. The prevalence increased with advancing age for all cSVD markers, except PVS in the centrum semiovale. The prevalence of moderate-to-severe deep WMHs was higher in women than in men (*P* = 0.005). Older age and hypertension were associated with increased likelihoods of all cSVD markers. A higher body mass index was linked to more WMHs. Coronary heart disease (CHD) was associated with WMHs, CMBs, and lacunes. Our study suggests that cSVD, especially WMHs and PVS, was highly prevalent among rural Chinese older adults. Older age, hypertension, and CHD are associated with distinct cSVD. Future prospective cohort studies are warranted to determine incidence and major risk factors of cSVD, which could facilitate preventive interventions to reduce the burden of cSVD in resource-limited settings.

## Teaser

Zhang *et al*. report that various markers of cerebral small vessel disease were highly prevalent among rural-dwelling Chinese older adults. Older age, hypertension, and coronary heart disease were the main factors associated with cerebral small vessel disease.

## Graphical Abstract

**Graphical Abstract:**
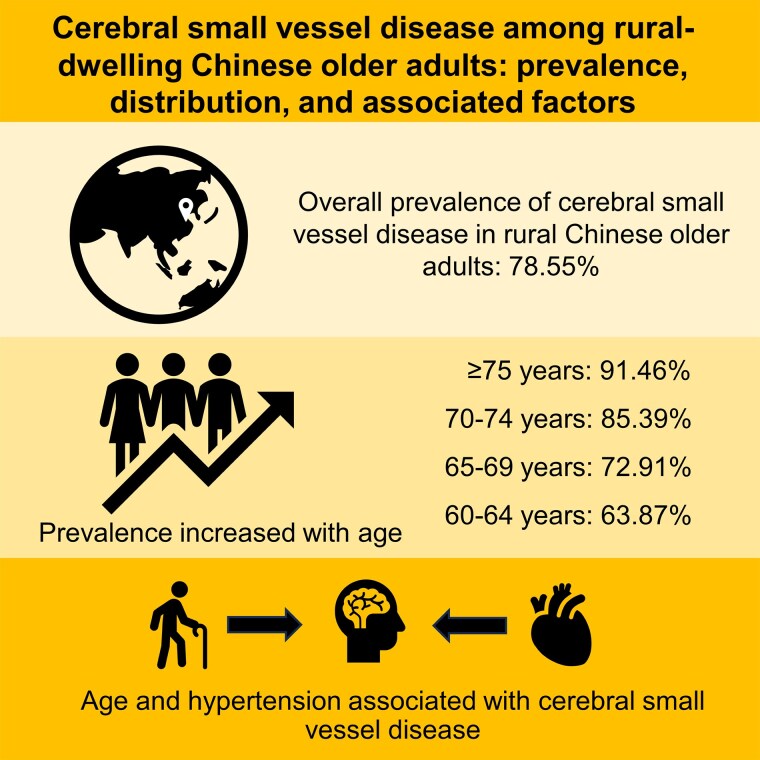

## Introduction

Cerebral small vessel disease (cSVD) refers to a variety of pathological processes that affect the small arteries, arterioles, venules and capillaries of the brain.^1^ Cerebral SVD can be detected using structural brain MRI technique, characterized by MRI markers such as white matter hyperintensities (WMHs), cerebral microbleeds (CMBs), lacunes and perivascular spaces (PVS). In addition, a composite summary score that integrates various cSVD markers has been proposed in previous studies to reflect burden of cSVD.^2-4^ As people age, cSVD becomes increasingly common, affecting around 5% of people aged 50 years to nearly 100% of people over 90 years of age.^5^ It has been well established that cSVD is associated not only with clinical stroke and dementia but also with mobility, mortality, neurobehavioural and mood disorders in older adults.^2,4,6^

The semi-systematic review of population-based studies, mostly from urban populations in high-income countries, reported that the prevalence ranges from 3.1 to 15.3% for CMBs, from 8 to 31% for lacunes, and from 65 to 96% for WMHs.^7^ The systematic review of community-based studies from low- and middle-income countries suggested that the median prevalence of cSVD was 10.7% for CMBs, 0.8% for lacunes, 20.5% for moderate-to-severe WMHs, and 25.0% for moderate-to-severe PVS.^8^ Notably, data on the prevalence and risk factors of cSVD from the general population setting in China, especially rural older adults, are currently limited. This is important because rural and urban populations across countries and ethnicities have distinct socioeconomic status, lifestyle factors, genetic predispositions that could affect occurrence and distribution of cSVD.

Cerebral SVD reflects diverse pathological processes with distinct manifestations on conventional structural brain MRI. Arteriolosclerosis and cerebral amyloid angiopathy (CAA) are the most common pathologies involving cSVD.^1^ Arteriolosclerosis is strongly associated with older age, diabetes, and hypertension in particular.^1^ CAA is associated with older age and apolipoprotein E (*APOE*) ɛ4 allele, but not convincingly with hypertension or other traditional cardiovascular risk factors.^9^ Investigating lifestyle (e.g. physical activity, smoking and alcohol consumption) and clinical factors (e.g. obesity, hypertension and diabetes) associated with various cSVD markers within a rural Chinese older population may help better understand the etiopathophysiological mechanisms underlying different cSVD, and thus, highlighting potential targets for preventive interventions.

Therefore, in this population-based cross-sectional study, we sought (1) to describe prevalence and distributions of various MRI markers for cSVD, and further, (2) to explore lifestyles, clinical factors, and *APOE* genotypes associated with different cSVD markers among rural-dwelling Chinese older adults.

## Materials and methods

### Study design and participants

**Figure 1:**
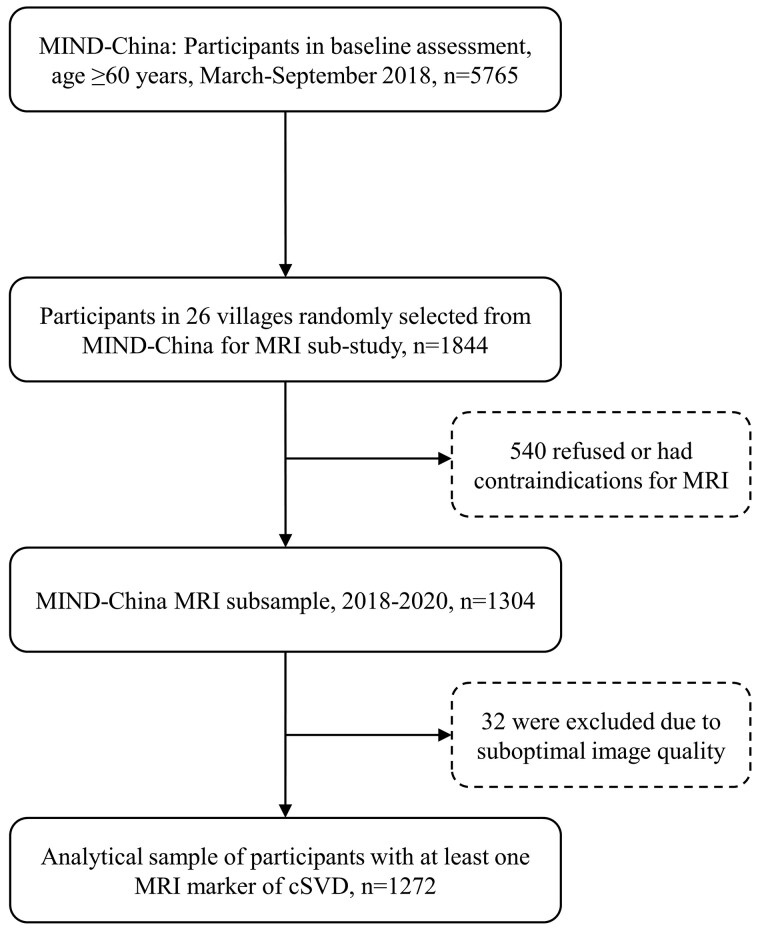
**Flowchart of study participants.** cSVD, cerebral small vessel disease; MIND-China, Multimodal INterventions to delay Dementia and disability in rural China; MRI, magnetic resonance imaging.

This was a population-based cross-sectional study. The study participants were derived from the MRI sub-study of the Multimodal Interventions to Delay Dementia and Disability in Rural China (MIND-China), a participating project of the World-Wide FINGERS Network.^10^ The MIND-China protocol was reviewed and approved by the ethics committee at Shandong Provincial Hospital in Jinan, Shandong. Written informed consent was obtained from all participants, or in the case of cognitively impaired persons, from a proxy (e.g. a family member). The baseline examination of the MIND-China Study targeted people who were aged ≥60 years and living in the 52 villages of Yanlou town, Yanggu County, western Shandong province, China, as previously reported.^11^ Briefly, in March-September 2018, 5765 (74.9% of all eligible) residents were examined for MIND-China. Of these, a subsample of 1844 participants from 26 villages that were randomly selected from all 52 villages in local Yanlou Town were invited to participate in the structural brain MRI sub-study from August 2018 to November 2020. Out of these, 1304 eventually agreed and undertook structural brain MRI scans. Compared with persons who did not undertake brain MRI scans (*n* = 4461), those who did (*n* = 1304) were slightly younger (mean age, 69.44 versus 71.31 years, *t* = 12.293, *P* < 0.001), and more educated (middle school or above, 19.91% versus 16.87%, χ² = 21.867, *P* = 0.013), but the two groups did not differ significantly in the distribution of sex (females, 58.44% versus 56.84%, χ² = 0.991, *P* = 0.320). In addition, among individuals who were invited to participate in the MIND-China MRI sub-study (*n* = 1844), there were no significant differences in age (mean age, 69.44 versus 69.22 years, *t* = 0.996, *P* = 0.319), sex (females, 58.44% versus 57.41%, χ² = 0.126, *P* = 0.722), or educational level (middle school or above, 19.91% versus 20.00%, χ² = 1.922, *P* = 0.382) between people who underwent brain MRI scans (*n* = 1304) and those who did not (*n* = 540) (Supplementary Table 1). Of the 1304 persons who undertook the brain MRI scans, 32 were excluded due to suboptimal image quality, leaving 1272 participants for the current analyses. Figure 1 shows a flowchart of the study participants.

### Data collection and assessment

Data collection and assessments have been fully reported elsewhere.^11^ In brief, we collected the following data via in-person interviews, clinical and neurological examinations, neuropsychological testing, and laboratory tests: demographic factors (age, sex, education, etc.), lifestyle habits (smoking, alcohol drinking, etc.), and medical history (hypertension, diabetes mellitus, dyslipidemia, etc.). Data on leisure-time physical activity were collected using a structured questionnaire that included different types of physical activities such as walking, sports, or other recreational pursuits. Physical inactivity was defined as the frequency of participating in physical activity less than once a week. Smoking and alcohol consumption status were categorized as ‘never’ versus ‘former or current’ smoking or drinking alcohol. Hypertension was defined as systolic blood pressure ≥140 mmHg or diastolic blood pressure ≥90 mmHg or current treatment with antihypertensive medication. Then, we further categorized hypertension into poorly controlled hypertension (blood pressure ≥140/90 mmHg) and well controlled hypertension (blood pressure <140/90 mmHg). Diabetes mellitus was defined as having self-reported history of diabetes ascertained by a physician or use of blood glucose-lowering medication or insulin injection or fasting serum glucose ≥7.0 mmol/L. Dyslipidemia was defined as fasting serum total cholesterol ≥6.2 mmol/L or triglycerides ≥2.3 mmol/L or low-density lipoprotein cholesterol ≥4.1 mmol/L or high-density lipoprotein cholesterol <1.0 mmol/L or use of lipid-lowering drugs. Stroke history was ascertained via in-person interviews and neurological examination. Dementia was clinically diagnosed according to the Diagnostic and Statistical Manual of Mental Disorders, Fourth Edition, criteria, and a 3-step diagnostic procedure was used, as previously reported.^12,13^

### MRI acquisition and assessments of cSVD markers

The brain MRI scans for all eligible participants were performed either on the Philips Ingenia 3.0T MR System (Philips Healthcare, Best, The Netherlands) in Southwestern Lu Hospital (*n* = 1178) or on the Philips Archiva 3.0T MR System (Philips Healthcare, Best, The Netherlands) in Liaocheng People’s Hospital (*n* = 126). Sagittal 3D T1-weighted structural images, axial T2-weighted images, 3D fluid-attenuated inversion recovery (FLAIR) images, and axial susceptibility-weighted imaging (SWI) sequences were acquired. The detailed parameters of these MRI sequences were previously reported.^11^

We used the AccuBrain® (BrainNow Medical Technology Ltd., Shenzhen, Guangdong, China) to assess CMBs, as described previously.^14^ Briefly, CMBs were detected on SWI images via a fully connected network that was trained by deep learning technique. For a given SWI image, the network showed CMB location by exporting a probability map. Then, a CMB segmentation image was produced based on the optimal threshold chosen in the training process. CMBs were categorized by locations into lobar CMBs (frontal, parietal, temporal, occipital, cingulate lobe and insula), deep CMBs (basal ganglia, thalamus, internal capsule, corpus callosum and deep white matter), and infratentorial CMBs (brainstem and cerebellum). The T2 FLAIR images were processed in AccuBrain to automatically acquire WMHs volume, as previously reported.^15^ In brief, the AccuBrain used T2 FLAIR images to calculate the signal contrast between normal brain tissue and WMHs and set the signal threshold to recognize WMHs.^16^ Based on predefined threshold, WMHs were recognized and extracted on T2 FLAIR images. Finally, the AccuBrain refined and localized WMHs using the transformed T1-weighted brain structure mask extracted from our study sample.

Lacunes, WMHs, PVS were then rated visually in accordance with the Standards for Reporting Vascular Changes on Neuroimaging-1 criteria.^17^ Briefly, lacunes were defined as fluid-filled cavities of 3–15 mm in diameter, with a CSF-like hypointensity surrounded by a rim of hyperintensity on FLAIR images. Deep WMHs (DWMHs) and periventricular WMHs (PWMHs) were visually graded separately on FLAIR images according to Fazekas scale.^18^ The presence of moderate-to-severe DWMHs or PWMHs was defined as the Fazekas score ≥2. PVS was defined as round, ovoid, or linear lesions of <3 mm size with a signal similar to that of CSF without a surrounding hyperintense rim. PVS was graded in the slice that contained the maximum number of PVS in the basal ganglia or the centrum semiovale, and in case of any asymmetry, PVS number of the worse side was used.^19^ The presence of PVS was defined as PVS count >10^3,20^ A summary score that had been validated in previous studies was used to summarize the global cSVD burden.^2,3^ One point was assigned to the summary score for the presence of (a) lacunes; (b) CMBs; (c) BG-PVS >10; and (d) DWMHs (Fazekas score 2–3) or PWMHs (Fazekas score 3). The summary score for global cSVD ranges from 0 to 4, and the presence of global cSVD was defined as the summary score ≥1.^21^

The trained rater (M.Z., a junior neurologist), who was blinded to the clinical data, visually assessed CMBs in 100 randomly selected subjects and then counted the BG-PVS, CSO-PVS bilaterally under the supervision of a senior clinical neurologist (L.S.). Three months after the initial assessment, PVS were re-assessed in MRI images of 30 randomly selected subjects, which yielded the intraclass correlation coefficient of 0.893 for the BG-PVS and 0.831 for the CSO-PVS. The κ statistic was 0.702 for the agreement between the presence of CMBs assessed visually and automatically. Lacunes and WMHs were assessed by a trained rater (J.W., a junior neurologist) under the supervision of a senior neurologist (L.S.) and an experienced neuroradiologist (T.G.). Six months after the initial assessment, the rater re-assessed MRI images of 200 randomly selected subjects for lacunes and WMHs, which yielded a weighted κ statistic of 0.838 for lacunes, 0.856 for DWMHs, and 0.892 for PWMHs.^22^

### Statistical analysis

Characteristics of the study participants by the presence of global cSVD were compared using Mann-Whitney U test for continuous variables and chi-square test for categorical variables. We reported the crude prevalence rates of various cSVD markers. We used the age- and sex-structures of rural population from the Seventh China National Population Census (2020) (www.stats.gov.cn/sj/pcsj) to standardize the overall prevalence of cSVD with direct method. We presented the age- and sex-specific prevalence rates of MRI markers for cSVD. Then, we used logistic or linear regression model to estimate the odds ratio or β and 95% confidence interval of various cSVD markers associated with lifestyles, clinical conditions, and *APOE* genotype while controlling for age, sex, education, and stroke history. Statistical significance was set at two-tailed *P* < 0.05. R 4.4.3 (RStudio: Integrated Development for R. RStudio PBC, Boston, MA) was used for all the analyses.

## Results

### Characteristics of study participants

Of the 1272 participants in the analytical sample, the mean age was 69.43 years (SD, 4.31), 58.57% were women, 35.22% received no formal schooling education, 81.15% were farmers, and 67.51% had hypertension, with 83.68% of those with hypertension being poorly controlled in their blood pressure. The presence of global cSVD was defined in 989 (78.55%) of all the participants. Compared with participants without global cSVD (*n* = 270), those with global cSVD were older, more educated, more likely to have hypertension, and had a higher prevalence of stroke and dementia (Table 1). The two groups did not differ significantly in the distribution of sex, occupation, physical inactivity, BMI, smoking, alcohol consumption, diabetes mellitus, dyslipidemia, coronary heart disease (CHD), or *APOE* ɛ4 allele (*P* > 0.05).

**Table 1:** Characteristics of the study participants in the total sample and by the presence of global cSVD

	Total sample	Presence of global cSVD (*n* = 1259)^a^		
Characteristics	(*n* = 1272)	No (*n* = 270)	Yes (*n* = 989)	*P*-value*
Age (years), mean (SD)	69.43 (4.31)	67.76 (3.85)	69.91 (4.32)	<0.001
Age groups (years), *n* (%)				<0.001
60–64	158 (12.42)	56 (20.74)	99 (10.01)	
65–69	508 (39.94)	136 (50.37)	366 (37.01)	
70–74	442 (34.75)	64 (23.70)	374 (37.82)	
≥75	164 (12.89)	14 (5.19)	150 (15.17)	
Female sex, *n* (%)	745 (58.57)	160 (59.26)	575 (58.14)	0.794
No formal education, *n* (%)	448 (35.22)	113 (41.85)	332 (33.57)	0.014
Farmers, *n* (%)	1029 (81.15)	228 (84.44)	789 (80.10)	0.127
Physical inactivity, *n* (%)	368 (29.00)	88 (32.59)	276 (27.99)	0.161
BMI (kg/m²), mean (SD)	24.97 (3.51)	24.59 (3.24)	25.09 (3.57)	0.110
Smoking, *n* (%)				0.805
Never smoking	826 (64.94)	177 (65.56)	638 (64.51)	
Ever smoking	446 (35.06)	93 (34.44)	351 (35.49)	
Alcohol consumption, *n* (%)				0.886
Never drinking	779 (61.68)	167 (62.08)	602 (61.37)	
Ever drinking alcohol	484 (38.32)	102 (37.92)	379 (38.63)	
Clinical conditions, *n* (%)				
Hypertension				<0.001
No	413 (32.49)	126 (46.67)	281 (28.41)	
Yes, well controlled	140 (11.01)	37 (13.70)	100 (10.11)	
Yes, poorly controlled	718 (56.49)	107 (39.63)	608 (61.48)	
Diabetes mellitus	191 (15.02)	39 (14.44)	149 (15.07)	0.875
Dyslipidemia	311 (24.45)	66 (24.44)	241 (24.37)	1.000
CHD	232 (18.24)	43 (15.93)	186 (18.81)	0.318
Stroke	160 (12.58)	19 (7.04)	139 (14.05)	0.003
Dementia	28 (2.22)	1 (0.37)	26 (2.65)	0.042
* APOE* ɛ4 allele	188 (15.03)	38 (14.45)	146 (14.96)	0.913

*APOE*, apolipoprotein E gene; BMI, body mass index; CHD, coronary heart disease; cSVD, cerebral small vessel disease.

^a^Of the 1272 participants in the total analytical sample, 13 were excluded from the analysis of global cSVD due to missing data on at least one of the four cSVD markers necessary for generating the global cSVD measure.

^*^ *P*-value was for the test of differences between the presence and absence of global cSVD.

### Prevalence and distributions of cSVD

The crude prevalence of CMBs, lacunes, global WMHs, DWMHs, PWMHs, global PVS, BG-PVS, CSO-PVS and global cSVD were 20.31, 26.87, 98.35, 95.74, 94.17, 86.61, 60.06, 76.31 and 78.55%, respectively (Table 2). After standardization using the 2020 China National Census data for rural population, the age- and sex-standardized prevalence rates of various cSVD markers slightly decreased (Table 2), with the prevalence being decreased by 6.75% for CMBs, 2.53% for lacunes, 1.25% for PWMHs, and 1.18% for global cSVD.

**Table 2:** Crude and age- and sex-standardized prevalence (per 100 population) of cSVD

MRI markers of cSVD	No. of subjects	No. of cases	Crude prevalence (95% CI), per 100 population	Standardized prevalence (95% CI), per 100 population^a^
CMBs	1211	246	20.31 (18.05–22.58)	18.94 (16.73–21.15)
Strictly lobar CMBs	1211	56	4.62 (3.44–5.81)	5.09 (3.85–6.33)
Strictly deep or infratentorial CMBs	1211	133	10.98 (9.22–12.74)	9.36 (7.72–11.01)
Mixed CMBs	1211	57	4.71 (3.51–5.90)	4.48 (3.32–5.65)
Lacunes	1269	341	26.87 (24.43–29.31)	26.19 (23.77–28.61)
Global WMHs	1269	1248	98.35 (97.64–99.05)	98.20 (97.47–98.93)
DWMHs	1269	1215	95.74 (94.63–96.86)	95.71 (94.60–96.83)
PWMHs	1269	1195	94.17 (92.88–95.46)	92.99 (91.59–94.40)
Global PVS	1262	1093	86.61 (84.73–88.49)	86.20 (84.29–88.10)
BG-PVS	1262	758	60.06 (57.36–62.77)	59.70 (57.00–62.41)
CSO-PVS	1262	963	76.31 (73.96–78.65)	76.06 (73.70–78.41)
Global cSVD	1259	989	78.55 (76.29–80.82)	77.62 (75.32–79.93)

BG-PVS, basal ganglia perivascular spaces; CMBs, cerebral microbleeds; CSO-PVS, centrum semiovale perivascular spaces; cSVD, cerebral small vessel disease; DWMHs, deep white matter hyperintensities; PVS, perivascular spaces; PWMHs, periventricular white matter hyperintensities; WMHs, white matter hyperintensities.

^a^The age- and sex-structures of rural population from the Seventh China National Population Census (2020) were used to standardize the overall prevalence of cSVD.

**Figure 2:**
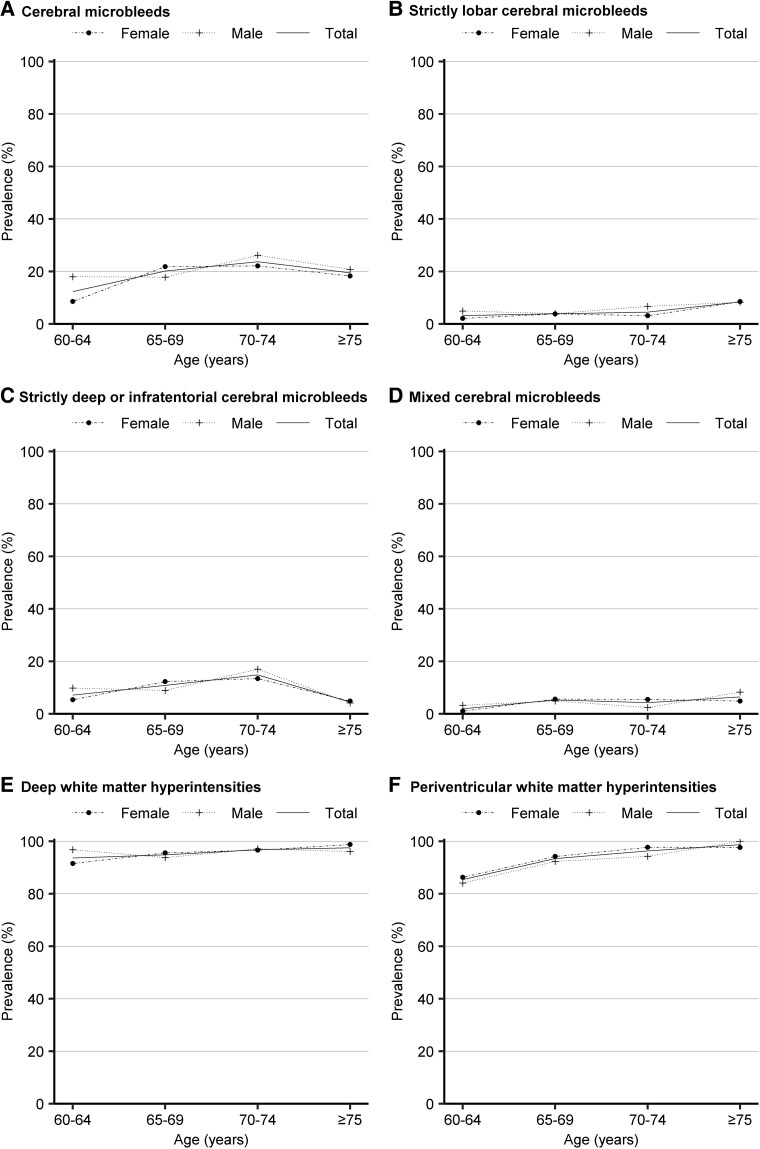
**The age- and sex-specific prevalence of CMBs (A), strictly lobar CMBs (B), strictly deep or infratentorial CMBs (C), mixed CMBs (D), DWMHs (E), and PWMHs (F).** The number of participants with missing values was 61 for CMBs and 3 for WMHs. The Fisher's exact test was used to compare the prevalence of strictly lobar CMBs in 60–64 age group, strictly deep or infratentorial CMBs in 60–64 and ≥75 age group, mixed CMBs in 60–64 and ≥75 age group, DWMHs in 60–64 and ≥75 age group, PWMHs in ≥75 age group. The chi-square test was used to compare the prevalence of other MRI markers for cSVD between sex across the age groups. There were no significant differences in prevalence of these MRI markers across the age groups (all *P* > 0.05). The sample sizes for various MRI markers are as follows: CMBs: total sample (*n* = 1211), males (*n* = 499) and females (*n* = 712); WMHs: total sample (*n* = 1269), males (*n* = 526) and females (*n* = 743).

**Figure 3:**
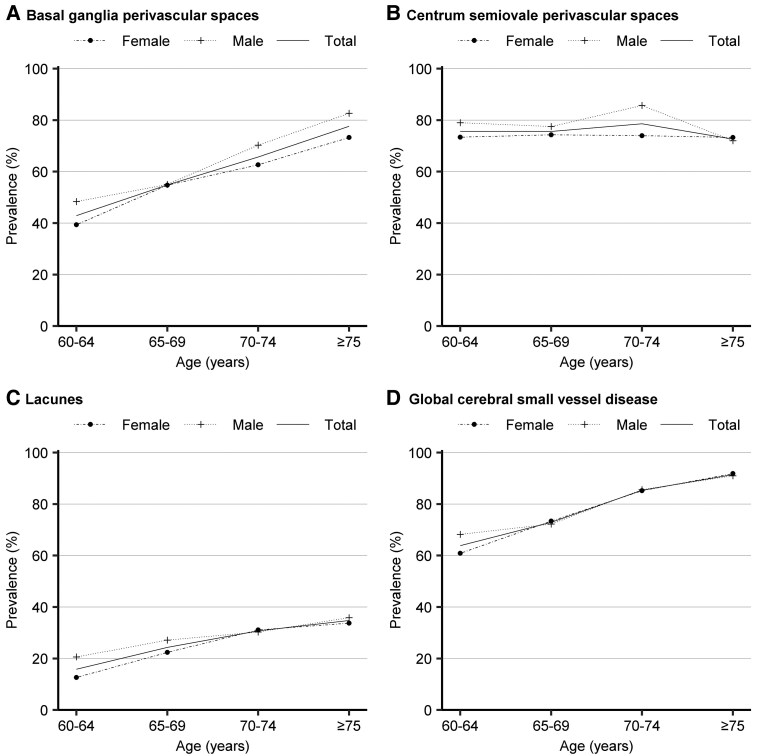
**The age- and sex-specific prevalence of BG-PVS (A), CSO-PVS (B), lacunes (C), and global cSVD (D).** Presence of global cSVD was defined as a summary score ≥1. The chi-square test was used to compare the prevalence of these MRI markers and global cSVD between sex across the age groups. In the 70–74 age group, the prevalence of CSO-PVS was significantly higher in males than females (χ² = 7.979, *P* = 0.005). There were no significant differences in prevalence of any other examined MRI markers for cSVD and global cSVD across the age groups (all *P* > 0.05). The sample sizes for various MRI markers and global cSVD are as follows: PVS: total sample (*n* = 1262), males (*n* = 521), and females (*n* = 741); Lacunes: total sample (*n* = 1269), males (*n* = 526) and females (*n* = 743); Global cSVD: total sample (*n* = 1259), males (*n* = 524) and females (*n* = 735).

Overall, the prevalence of all cSVD markers increased with age in both men and women, except for CSO-PVS, where the prevalence of CSO-PVS was slightly increased from 75.64% in people aged 60–64 years and those aged 65–69 years to 78.64% in those aged 70–74 years, and then decreased to 72.67% among individuals aged 75 years and above (Figs 2 and 3). The prevalence of all individual cSVD markers and global cSVD showed no significant sex difference across all age groups (*P* > 0.05), except that the prevalence of CSO-PVS in the age group of 70–74 years was higher in males than in females (*P* = 0.005).

### Factors associated with cSVD

First, of the demographic factors, the age-related increases in the overall prevalence of all MRI markers of cSVD, except CSO-PVS, were confirmed in the logistic regression analysis after controlling for sex, education and stroke history (Tables 3 and 4). There were no significant sex differences in overall prevalence of all cSVD markers, except moderate-to-severe DWMHs, where men had a lower likelihood than women. Having any education (versus no school education) was significantly associated with increased likelihoods of CSO-PVS and global cSVD after controlling for age, sex and stroke history. Being farmers was significantly associated with a lower likelihood of global cSVD (*P* = 0.049). Second, of the lifestyle-related factors, a higher BMI was significantly associated with a greater WMH volume and increased likelihoods of WMHs and global cSVD, but not with other examined MRI markers of cSVD. Third, out of the clinical factors, controlling for age, sex, education and stroke history, hypertension, especially poorly controlled, but not well controlled hypertension, was associated with increased likelihoods of global cSVD and all the examined MRI cSVD markers, except CSO-PVS and strictly lobar CMBs. In addition, having CHD was significantly associated with a greater WMH volume and increased likelihoods of global CMBs, mixed CMBs, and lacunes. Diabetes, dyslipidemia, and *APOE* ɛ4 allele were not significantly associated with any of the examined MRI markers of cSVD or global cSVD, except for the *APOE* ɛ4 allele, which was significantly associated with an increased likelihood of mixed CMBs (Tables 3 and 4).

**Table 3:** Associations of lacunes and CMBs with demographic, lifestyle, clinical factors, and APOE genotypes

	Odds ratio (95% CI), cSVD markers				
Factors	Lacunes	Any CMBs	Strictly lobar CMBs	Strictly deep or infratentorial CMBs	Mixed CMBs
**Demographic factors**					
Age, years	1.07 (1.04–1.10)^c^	1.04 (1.00–1.07)^a^	1.05 (0.99–1.12)	1.02 (0.98–1.06)	1.04 (0.98–1.11)
Male sex	1.02 (0.77–1.36)	1.14 (0.82–1.57)	1.43 (0.78–2.63)	1.16 (0.76–1.77)	0.80 (0.44–1.46)
Any education	1.31 (0.97–1.78)	0.87 (0.62–1.21)	1.01 (0.52–1.95)	0.72 (0.47–1.10)	1.24 (0.66–2.33)
Farmers	0.92 (0.65–1.30)	0.90 (0.61–1.32)	0.59 (0.30–1.14)	1.09 (0.65–1.82)	1.05 (0.49–2.24)
**Lifestyle factors**					
Physical inactivity	0.91 (0.68–1.21)	1.08 (0.79–1.47)	0.89 (0.47–1.66)	1.29 (0.87–1.91)	0.82 (0.44–1.53)
BMI (kg/m²)	1.03 (0.99–1.07)	1.02 (0.98–1.06)	0.96 (0.89–1.04)	1.05 (0.99–1.10)	1.02 (0.94–1.10)
Ever smoking	1.06 (0.67–1.69)	0.80 (0.49–1.33)	0.95 (0.38–2.35)	0.70 (0.37–1.31)	1.10 (0.39–3.07)
Ever drinking alcohol	0.95 (0.65–1.41)	0.81 (0.52–1.24)	0.67 (0.31–1.48)	0.77 (0.44–1.35)	1.23 (0.54–2.79)
**Clinical conditions**					
Hypertension					
No	1.00 (reference)	1.00 (reference)	1.00 (reference)	1.00 (reference)	1.00 (reference)
Yes	1.57 (1.18–2.09)^b^	1.70 (1.23–2.36)^b^	1.76 (0.92–3.38)	1.35 (0.90–2.03)	2.20 (1.09–4.41)^a^
Yes, well controlled	0.77 (0.47–1.27)	0.80 (0.45–1.44)	1.41 (0.52–3.86)	0.38 (0.15–1.00)^a^	1.67 (0.59–4.71)
Yes, poorly controlled	1.77 (1.32–2.37)^c^	1.90 (1.37–2.65)^c^	1.83 (0.94–3.55)	1.56 (1.03–2.35)^a^	2.30 (1.14–4.66)^a^
Diabetes mellitus	1.34 (0.95–1.89)	1.29 (0.88–1.88)	0.86 (0.38–1.94)	1.30 (0.81–2.10)	1.51 (0.78–2.94)
Dyslipidemia	1.06 (0.79–1.43)	0.78 (0.55–1.11)	1.13 (0.60–2.13)	0.76 (0.48–1.19)	0.67 (0.34–1.32)
CHD	1.79 (1.31–2.45)^c^	1.61 (1.15–2.27)^b^	0.87 (0.42–1.81)	1.49 (0.97–2.30)	2.27 (1.27–4.06)^b^
*APOE* ɛ4 allele	0.97 (0.68–1.39)	1.19 (0.81–1.75)	0.97 (0.45–2.10)	0.91 (0.53–1.53)	2.05 (1.09–3.86)^a^

Odds ratios (95% confidence intervals) were derived from models that were adjusted for age, sex, education, and stroke history.

*APOE*, apolipoprotein E gene; BMI, body mass index; CHD, coronary heart disease; CMBs, cerebral microbleeds; cSVD, cerebral small vessel disease.

^a^: *P* < 0.05, ^b^: *P* < 0.01, ^c^: *P* < 0.001.

**Table 4:** Associations of WMHs, PVS and global cSVD burden with demographic, lifestyle, clinical factors, and APOE genotypes

	β (95% CI)	Odds ratio (95% CI)				
Factors	Global WMHs volume^a^	Moderate-to-severe DWMHs	Moderate-to-severe PWMHs	BG-PVS	CSO-PVS	Global cSVD
**Demographic factors**						
Age, years	0.06 (0.05–0.07)^d^	1.07 (1.04–1.10)^d^	1.13 (1.09–1.16)^d^	1.11 (1.08–1.14)^d^	0.99 (0.96–1.02)	1.13 (1.09–1.16)^d^
Male sex	0.04 (−0.07–0.15)	0.69 (0.54–0.90)^c^	0.83 (0.64–1.08)	1.17 (0.89–1.52)	1.20 (0.89–1.63)	0.86 (0.63–1.19)
Any education	0.02 (−0.1–0.13)	1.07 (0.82–1.39)	0.91 (0.69–1.19)	1.16 (0.88–1.52)	1.37 (1.01–1.85)^b^	1.42 (1.03–1.97)^b^
Farmers	0.01 (−0.12–0.15)	1.09 (0.80–1.48)	1.09 (0.79–1.50)	0.85 (0.61–1.18)	1.01 (0.69–1.46)	0.67 (0.45–1.00)^b^
**Lifestyle factors**						
Physical inactivity	0.03 (−0.08–0.13)	1.18 (0.92–1.52)	1.07 (0.83–1.38)	0.81 (0.63–1.05)	0.91 (0.68–1.21)	0.91 (0.68–1.23)
BMI (kg/m²)	0.02 (0.01–0.04)^c^	1.05 (1.01–1.08)^c^	1.04 (1.00–1.07)^b^	1.03 (0.99–1.06)	1.01 (0.97–1.05)	1.06 (1.02–1.10)^c^
Ever smoking	−0.07 (−0.25–0.11)	0.79 (0.52–1.19)	1.00 (0.65–1.53)	1.15 (0.75–1.77)	0.75 (0.45–1.25)	1.03 (0.61–1.72)
Ever drinking alcohol	−0.07 (−0.22–0.08)	0.71 (0.5–1.00)	0.78 (0.55–1.12)	1.11 (0.77–1.59)	0.85 (0.56–1.28)	1.06 (0.69–1.65)
**Clinical conditions**						
Hypertension						
No	0.00 (reference)	1.00 (reference)	1.00 (reference)	1.00 (reference)	1.00 (reference)	1.00 (reference)
Yes	0.27 (0.16–0.37)^d^	1.37 (1.07–1.74)^b^	1.58 (1.24–2.03)^d^	1.89 (1.47–2.41)^d^	1.27 (0.97–1.68)	2.12 (1.59–2.82)^d^
Yes, well controlled	0.06 (−0.1–0.23)	0.90 (0.61–1.34)	1.10 (0.74–1.64)	1.18 (0.79–1.76)	1.17 (0.74–1.84)	1.04 (0.67–1.63)
Yes, poorly controlled	0.31 (0.2–0.41)^d^	1.48 (1.15–1.90)^c^	1.70 (1.32–2.20)^d^	2.08 (1.61–2.68)^d^	1.30 (0.98–1.72)	2.50 (1.85–3.38)^d^
Diabetes mellitus	0.02 (−0.12–0.15)	0.96 (0.70–1.31)	1.22 (0.88–1.69)	0.93 (0.67–1.28)	0.98 (0.68–1.42)	1.07 (0.72–1.58)
Dyslipidemia	−0.01 (−0.12–0.11)	1.10 (0.84–1.43)	1.23 (0.94–1.62)	1.06 (0.80–1.39)	0.96 (0.70–1.30)	0.98 (0.70–1.36)
CHD	0.17 (0.04–0.29)^c^	1.30 (0.97–1.75)	1.12 (0.83–1.51)	1.13 (0.83–1.53)	1.11 (0.79–1.56)	1.09 (0.75–1.58)
*APOE* ɛ4 allele	−0.08 (−0.22–0.05)	0.79 (0.58–1.09)	0.96 (0.70–1.33)	0.97 (0.70–1.35)	0.82 (0.57–1.17)	1.04 (0.70–1.55)

β coefficients or odds ratios (95% confidence intervals) were derived from models that were adjusted for age, sex, education, and stroke history.

^a^Global WMHs volume was transformed as ln (WMHs volume + 1).

*APOE*, apolipoprotein E gene; BG-PVS, basal ganglia perivascular spaces; BMI, body mass index; CHD, coronary heart disease; CSO-PVS, centrum semiovale perivascular spaces; cSVD, cerebral small vessel disease; DWMHs, deep white matter hyperintensities; PWMHs, periventricular white matter hyperintensities.

^b^: *P* < 0.05, ^c^: *P* < 0.01, ^d^: *P* < 0.001.

## Discussion

In this population-based study of rural-dwelling older adults in China, we found a high prevalence of various MRI markers of cSVD, with WMHs being the most common cSVD, followed by PVS, lacunes and CMBs. Furthermore, our data showed that older age and hypertension were associated with different cSVD markers, whereas CHD was associated with CMBs, WMHs and lacunes. These findings contribute to the current literature regarding epidemiology (e.g. prevalence, distribution and related factors) of cSVD among a rural-dwelling older population in China.

The prevalence and distribution of cSVD varied substantially across studies depending on characteristics of the study populations and methodological issues in the detection and assessments of cSVD. Thus, variations in demographic characteristics, geographic regions, ethnicities, and health conditions of the study samples, as well as MRI protocols, rating scales, and diagnostic or defining criteria, should be taken into consideration when comparing the prevalence and distribution of cSVD across population-based studies.

The prevalence of CMBs in our study (20.3%, mean age 69.4 years) was comparable to that observed from the Rotterdam Scan Study (23.5%, mean age 69.6 years) in the Netherlands and Beijing Shunyi Study (20.0% for those ≥60 years) in China,^23,24^ but lower than the report from the US Multi-Ethnic Study of Atherosclerosis (MESA, 33%, mean age 72 years).^25^ The higher prevalence of CMBs in the MESA study may be partly attributed to differences in characteristics of study samples; for instance, the MESA study included multiracial participants and had older age, a greater BMI, a higher prevalence of diabetes, and a higher frequency of *APOE* ɛ4 allele. However, the prevalence of CMBs in our study sample was higher than that of the Icelandic AGES-Reykjavik Study (16.8%, mean age 74.6 years),^26^ partly due to the facts that our study used the higher field MRI scanners (3.0T versus 1.5T) and the more sensitive approach (deep learning versus manual assessment) to detect CMBs. The prevalence of lacunes in our study (26.9%) was similar to that in the Longitudinal Healthy Aging Brain (26.4%, mean age 70.8 years) in Switzerland, pooling data from three population-based studies in the Asia-Pacific region (24.6%, mean age 70.1 years), and the Beijing Shunyi Study (29.0%),^27,28^ but lower than the reports from the Tasmanian Study of Cognition and Gait (TASCOG, 34.2%, mean age 72.1 years) in Australia.^29^ This difference may be partly attributed to older age, the higher prevalence of hypertension and smoking, and a higher BMI among participants of the TASCOG Study. For WMHs and PVS, variations in rating scales and cut-off values for defining these MRI markers across studies make the direct comparison difficult. We reported a high prevalence of WMHs (98.4% for global WMHs, 95.7% for DWMHs and 94.2% for PWMHs). This was similar to the reports from the Rotterdam Scan Study (95%, mean age 72 years) and the US Cardiovascular Health Study (96%, mean age 74 years),^7,30,31^ but higher than an observational study that included patients who undertook the diagnostic brain MRI scans for different reasons (56.2%, mean age 65.96 years).^32^

The prevalence of PWMHs was similar to that of the PolyvasculaR Evaluation for Cognitive Impairment and Vascular Events (PRECISE, 99.4%, mean age 61.2 years) and the Beijing Shunyi Study (90.1%), while the prevalence of DWMHs was higher than that of PRECISE (86.7%) and the Beijing Shunyi Study (84.8%).^33^ This difference may partly be attributed to the higher prevalence of hypertension with very low control rates and the inclusion of participants with a history of stroke in our population. The prevalence of PVS in our sample (86.6% for global PVS, 60.1% for BG-PVS and 76.3% for CSO-PVS) was higher than that of most previous studies, particularly for BG-PVS. For instance, a community-study of older adults in Atahualpa (mean age 71.1 years) reported a prevalence of 31% for BG-PVS and the Sleep Heart Health Study in US (mean age: 63.0 years) reported a prevalence of 51.1% for CSO-PVS.^34,35^ Furthermore, the prevalence of PVS in our study sample was markedly higher than that in the PRECISE study (9.8% for BG-PVS and 37.6% for CSO-PVS). This difference may partly be attributed to the fact that high blood pressure was associated with PVS, especially BG-PVS^36,37^ and that hypertension was poorly controlled in the large majority of individuals with hypertension in our study. Finally, the prevalence of PVS in the Framingham Heart Study Original and Offspring cohort (54.9% for BG-PVS and 71.5% for CSO-PVS, mean age 68.9 years) and the Three-City Study in France (76.4% for CSO-PVS, mean age 72.5 years) was similar to our reports.^38,39^ Taken together, the differences in mean age, socioeconomic status, prevalence and control rate of hypertension, and health conditions (e.g. stroke and dementia) might partly contribute to the variations in prevalence of various cSVD markers across studies.

Our study showed that older age, hypertension, and poorly controlled hypertension in particular were consistently associated with various individual cSVD markers and the global cSVD measure. This has significant implications for prevention of cSVD in rural China given the facts that incidence of hypertension has increased since the 1990s^40^ and that hypertension remains highly prevalent and poorly controlled, especially among rural older adults.^41,42^ We also found that a higher BMI and CHD were associated with several cSVD markers (e.g. CMBs, WMHs and lacunes). Current evidence linking vascular risk factors (e.g. smoking, high BMI and dyslipidemia) with various cSVD markers remains mixed. Our study suggested that CSO-PVS and strictly lobar CMBs, which are likely due to CAA, were not associated with hypertension or other cardiovascular risk factors, which is in line with previous reports.^2,43,44^ By contrast, BG-PVS and mixed CMBs or deep CMBs, which largely reflect hypertensive arteriopathy,^45^ were associated with hypertension in our sample. Previous studies have shown that high blood pressure is associated with BG-PVS, but not evidently with CSO-PVS.^46^ We observed sex differences in prevalence of moderate-to-severe DWMHs, with the prevalence being higher in females than in males, which was consistent to some extent with previous studies that showed a higher burden and faster progression of DWMHs in older females than males.^7,47^ Differences in sex hormones or susceptibility to vascular risk factors may partly contribute to sex variations in prevalence of some cSVD markers.^48^

Our community-based MRI study involved a relatively large sample of older adults who were living in rural areas of China and who received no or very limited formal education and had relatively low socioeconomic status, distinct lifestyles, and limited access to healthcare services, a sociodemographic group that had been substantially underrepresented in current research of epidemiology of cSVD. Thus, findings from our study could partly bridge the gap in knowledge regarding epidemiology of cSVD in rural Chinese older populations and may potentially contribute to minimising the rural-urban disparities in brain health of older adults. In addition, we used 3.0T scanners and international criteria to detect and assess cSVD markers. However, our study does have certain limitations. Firstly, the cross-sectional nature of the study prevents us from establishing causality for any of the observed associations. Additionally, the analytical sample was relatively younger and healthier compared with the MIND-China total sample, which may potentially lead to an underestimation of the true prevalence and the associations of cSVD markers with various factors. Finally, our study sample was drawn from rural areas in western Shandong province, characterized by farmers, very limited education, and low socioeconomic status, which should be considered when generalising our findings to broader populations, even rural populations in China.

## Conclusion

Our population-based study revealed that cSVD markers were highly prevalent among rural-dwelling older adults in China. Older age, hypertension, and CHD were the main factors associated with various cSVD markers. While findings from our study could address the knowledge gap between rural and urban populations with regard to epidemiology of cSVD, future prospective cohort studies are imperative to determine the incidence and risk factors for the occurrence and progression of various cSVD markers, which may pave the way for preventive and therapeutic interventions to delay cSVD in older people and promote healthy brain aging.

## Supplementary Material

fcaf136_Supplementary_Data

## Data Availability

The data are available from the corresponding authors upon reasonable request.
