# Supplementary material for: Cerebral small vessel disease among rural-dwelling Chinese older adults: prevalence, distribution, and associated factors
Source: Brain Commun. 2025 Apr 4;7(2):fcaf136. doi: 10.1093/braincomms/fcaf136 (PMC12006717; doi:10.1093/braincomms/fcaf136)
Supplement: fcaf136_Supplementary_Data [file fcaf136_supplementary_data.docx]

Supplementary Table 1, Characteristics between the invited persons who participated in the brain MRI scans and those who did not

| **Characteristics** | **Included (n=1304)** | **Excluded (n=** **540)** | **P value** |
| --- | --- | --- | --- |
| Age (years), mean (SD) | 69.44(4.29) | 69.22(4.48) | 0.319 |
| Female, n (%) | 762 (58.44) | 310 (57.41) | 0.722 |
| Educational level, n (%) |  |  | 0.382 |
| Illiterate (no school education) | 454(35.03) | 206(38.15) |  |
| Primary school | 584(45.06) | 226(41.85) |  |
| Middle school and above | 258(19.91) | 108(20.00) |  |
| Smoking, n (%) |  |  | 0.872 |
| Never smoking | 838 (64.66) | 352 (65.19) |  |
| Current or former smoking | 458 (35.34) | 188 (34.81) |  |
| Alcohol consumption, n (%) |  |  | 0.912 |
| Never drinking | 793 (61.62) | 325 (61.21) |  |
| Current or former drinking | 494 (38.38) | 206 (38.79) |  |
| Body mass index (kg/m^2^), mean (SD) | 24.98(3.50) | 24.91(3.63) | 0.703 |
| Hypertension, n (%) | 868 (67.71) | 337 (62.52) | 0.038 |
| Diabetes, n (%) | 196(15.12) | 77(14.26) | 0.688 |
| Dyslipidemia, n (%) | 314(24.23) | 119(22.04) | 0.343 |
| Coronary heart disease, n (%) | 237(18.29) | 108(20.00) | 0.429 |
| Stroke, n (%) | 166(12.81) | 55(10.19) | 0.135 |
